# Functional Analyses of Flavonol Synthase Genes From *Camellia sinensis* Reveal Their Roles in Anther Development

**DOI:** 10.3389/fpls.2021.753131

**Published:** 2021-10-01

**Authors:** Yufeng Shi, Xiaolan Jiang, Linbo Chen, Wei-Wei Li, Sanyan Lai, Zhouping Fu, Yajun Liu, Yumei Qian, Liping Gao, Tao Xia

**Affiliations:** ^1^State Key Laboratory of Tea Plant Biology and Utilization, Anhui Agricultural University, Hefei, China; ^2^Tea Research Institute, Yunnan Academy of Agricultural Sciences, Yunnan Engineering Research Center of Tea Germplasm Innovation and Matching Cultivation, Menghai, China; ^3^School of Life Sciences, Anhui Agricultural University, Hefei, China; ^4^School of Biological and Food Engineering, Suzhou University, Suzhou, China

**Keywords:** *Camellia sinensis*, flower development, flower sterility, flavonols, flavonol synthase

## Abstract

Flavonoids, including flavonol derivatives, are the main astringent compounds of tea and are beneficial to human health. Many researches have been conducted to comprehensively identify and characterize the phenolic compounds in the tea plant. However, the biological function of tea flavonoids is not yet understood, especially those accumulated in floral organs. In this study, the metabolic characteristics of phenolic compounds in different developmental stages of flower buds and various parts of the tea flower were investigated by using metabolomic and transcriptomic analyses. Targeted metabolomic analysis revealed varying accumulation patterns of different phenolic polyphenol compounds during flowering; moreover, the content of flavonol compounds gradually increased as the flowers opened. Petals and stamens were the main sites of flavone and flavonol accumulation. Compared with those of fertile flowers, the content of certain flavonols, such as kaempferol derivatives, in anthers of hybrid sterile flowers was significantly low. Transcriptomic analysis revealed different expression patterns of genes in the same gene family in tea flowers. The *CsFLSb* gene was significantly increased during flowering and was highly expressed in anthers. Compared with fertile flowers, *CsFLSb* was significantly downregulated in sterile flowers. Further functional verification of the three *CsFLS* genes indicated that *CsFLSb* caused an increase in flavonol content in transgenic tobacco flowers and that *CsFLSa* acted in leaves. Taken together, this study highlighted the metabolic properties of phenolic compounds in tea flowers and determined how the three *CsFLS* genes have different functions in the vegetative and reproductive organs of tea plants. Furthermore, *CsFLSb* could regulated flavonol biosynthesis in tea flowers, thus influencing fertility. This research is of great significance for balancing the reproductive growth and vegetative growth of tea plants.

## Introduction

**FIGURE 1:**
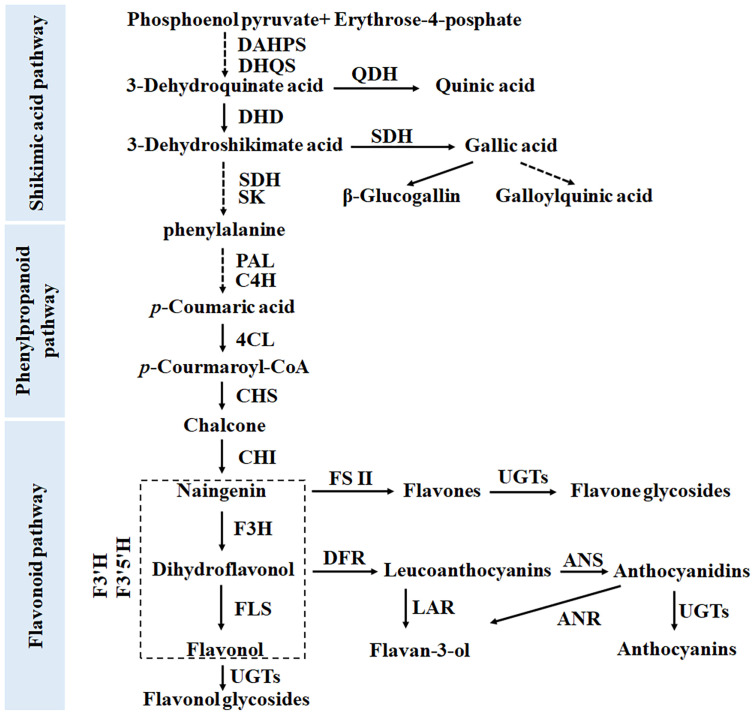
Biosynthetic pathway of phenolic compounds in tea plants, including the shikimate, phenylpropanoid, and flavonoid pathways.

Tea plant is a highly popular beverage crop cultivated in tropical and temperate regions around the world (Valduga et al., 2019). Tea plants are rich in polyphenols, caffeine, and amino acids, which are beneficial for human health, including antibacterial and anti-inflammatory properties, cancer prevention, and brain dysfunction suppression (Tang et al., 2019; Xing et al., 2019). Tea polyphenols include phenolic acids, catechins, anthocyanins, flavones, and flavonol and are synthesized through the shikimate, phenylpropanoid, and flavonoid pathways (Jiang et al., 2013; Figure 1). Polyphenols in help plants to resist biotic and abiotic stresses. For example, under drought conditions, the metabolic flux to the synthetic pathway of secondary metabolites, such as flavanols and anthocyanins, is upregulated (Jiang et al., 2017; Zahedi et al., 2019). In *Arabidopsis thaliana* and tobacco, the accumulation of anthocyanins is regulated, which yields improved tolerance to low temperature, drought, and salt stress (Li et al., 2017; Naing et al., 2018). Given their antioxidant effects, flavonols can help plants deal with stress by, for example, maintaining the steady state of reactive oxygen species (ROS) and thus protecting pollen from temperature stress (Muhlemann et al., 2018).

Flavonols, such as kaempferol (K) and quercetin (Q), can act as endogenous negative regulators of auxin transport, thereby affecting plant development (Jacobs and Rubery, 1988; Brown et al., 2001). These flavonoids can block the binding ability of the indole-3-acetic acid (IAA) polar transport inhibitor N-1-naphthylphthalamic acid and inhibit auxin movement in hypocotyl segments (Jacobs and Rubery, 1988; Wasson et al., 2006). The polar transport of auxin regulates the polar growth of plant tips, such as the root tip, the stem tip, filaments, and the pollen tube (Tansengco et al., 2004). Compared with wild-type strains, Arabidopsis mutants lacking flavonoids have fewer root hairs, shorter root hairs, and pollen tubes that exhibit abnormal growth (Schiefelbein et al., 1993). Knocking out the chalcone synthase (*CHS*) gene in petunias and silencing the expression of the flavonol synthase (*FLS*) gene in tobacco both reduce flavonol content, leading to blocked pollen tube germination and male sterility (Napoli et al., 1999; Mahajan et al., 2011). The addition of a low concentration of flavonols to the germination medium promotes pollen germination and pollen tube growth (Ylstra et al., 1992; Pollak et al., 1995).

The physiological significance of polyphenols in the tea plant is unclear. Exploration of the relationship between phenolic compounds and reproduction has practical significance. Tea plants have a long reproductive growth period, requiring 2 years from flowering to seed maturity. A competitive relationship exists between their reproductive and vegetative growth (Liu et al., 2020). Tea leaves are the raw material for making the beverage, however, flowering and seed production consume a large amount of nutrients contained in the leaf, thereby reducing the economic value of the tea plant. Therefore, flowers are regarded as industrial waste by tea farmers. However, for breeders, flowering is an essential physiological process of tea plants. Thus, determining the balance between the reproductive growth and vegetative growth of tea plants is a known challenge in the tea industry. To ensure the quality of tea beverage products in the following year, tea farmers usually take measures such as artificial pruning and spraying plant growth regulators to inhibit reproduction (Liu et al., 2019). Understanding the relationship between the development of flowers and secondary metabolites (such as polyphenols) in tea plants will provide new ideas for effectively controlling the reproductive growth of tea plants.

Although the tea flower is bisexual, it is self-incompatible (Seth et al., 2019); thus, cross-pollination is the main propagation method of tea plants. Although tea plants bloom numerous each year, the fruit-setting rate is only 2–14.13% (Dong, 2001). Changes in the levels of plant hormones, including ABA, IAA, and ethylene etc., are involved in regulating pollen–pistil interactions (Baker et al., 1997). As a second messenger, Ca2^+^ plays a key role in self-incompatibility (Wheeler et al., 2009). Many genes are involved in self-incompatibility, such as the *S* locus (S-locus) with multiple alleles (Takayama et al., 2000), *S-RNase* gene (Sun et al., 2018), *HT* gene, other modified genes (Kondo et al., 2002; Goldraij et al., 2006; Puerta et al., 2009), and *S-Locus F-box* (*SLF*) gene (Sun et al., 2015), which is involved in ubiquitin-mediated protein degradation (Hua and Kao, 2006).

It remains unclear how the phenolic compounds in flower buds change during flowering and whether they are involved in regulating the development of stamens and pistils. In this study, we used targeted metabolomic and transcriptomic analyses to evaluate the metabolism differences of phenolic compounds in buds at different developmental stages and in various parts of the tea flower. The key compounds and genes related to flower bud development was analyzed. These compounds and genes were then verified in fertile and hybrid sterile flower buds and transgenic tobacco.

## Materials and Methods

### Plant Materials

Cultivars of the tea plant “Shuchazao” were grown in an experimental tea field at Anhui Agricultural University, Hefei, China (East longitude 117.27, North latitude 31.86). The tea plant “Fudingbaicha” cultivars were grown in the Tea Plant Cultivar and Germplasm Resource Garden in Guohe Town, Anhui Agricultural University. The sterile flowers were obtained from the Tea Research Institute, Yunnan Academy of Agricultural Sciences/Yunnan Engineering Research Center of Tea Germplasm Innovation and Matching Cultivation, Menghai, China. For the convenience of comparison, the developmental stage of the flower was artificially divided into six stages (S1–S6) with significant differences in shape and size. The flower bud in stage 1 is extremely small, only 3.0–3.5 mm in diameter. The floral organ primordia have been previously initiated and differentiated (Zhang, 2012; Zhou et al., 2017). According to the cross-sectional view of the flower bud, in stage 3 of the flower bud, its petals begin to appear white and its anthers become bright yellow due to pigment accumulation. Second leaves and flowers at different developmental stages and different parts of the flower were collected and frozen immediately in liquid nitrogen and stored at −80°C. Live branches with flowers were kept fresh in absorbent sponge for subsequent experiments. Tobacco G28 (*Nicotiana tabacum* cv. G28) and transgenic tobacco were grown in a growth chamber at a constant temperature of 24 ± 3°C and 12/12 h light/dark cycle (Jiang et al., 2020).

### Metabolic Analysis of Phenolic Compounds in Tea and Tobacco Flowers

The total polyphenol content of the tea samples was extracted and identified as described by Jiang et al. (2013) and Zhuang et al. (2020) with some modifications: 50 mg (dry weight) of samples was extracted with 2 mL of 80% methanol solution. The parameters of fragmentor voltage and collision energy were optimized by Zhuang et al. (2020). The quantitative detection of the compounds was performed using the MRM mode of the Agilent 6460 QQQ-MS/MS LC system (Agilent Technologies, Palo Alto, CA, United States). The flavonol glycosides were extracted from tobacco flowers and quantified as proposed by Jiang et al. (2018, 2020).

### Transcriptome Analysis

The tissue samples used for transcriptome analysis were as follows: different developmental periods (stages 1, 3, and 5) of tea flowers of *Camellia sinensis cv. Shuchazao*. All samples were immediately frozen in liquid nitrogen and stored at −80°C. Each tissue had three biological repeats. On the basis of tea plants genome data, transcriptome sequencing and data analysis of tea flowers at stages 1, 3, and 5 were performed by BGI Gene Tech (Shenzhen, China) using the BGISEQ-500 platform.

Transcriptome data of different parts of flowers were obtained from the Tea Plant Information Archive (TPIA^1^). The data of *CsFLSb* expression in sterile and fertile flowers were obtained from transcriptomic data of the Yunnan Academy of Agricultural Sciences and Yunnan Engineering Research Center of Tea Germplasm Innovation and Matching Cultivation.

### Quantitative Real-Time PCR of Flavonoid Pathway Genes

Total RNA was isolated, and RNA quality and quantity were determined according to the method of Wang et al. (2018). First-strand cDNA synthesis was performed using PrimeScript RT Master Mix (Takara, DaLian, China). Two-Step real-time PCR assays were performed as described by Wang et al. (2018). The primer sequences used in this study are listed in Supplementary Table 3; they were selected following Wang et al. (2018). The data were expressed as the mean of three replicates.

### Genetic Transformation of *CsFLSs*

Tobacco genetic transformation was performed using the pCB2004-*Agrobacterium tumefaciens* expression system. The specific transgenic methods for tobacco were described by Jiang et al. (2020), and genetically modified materials were also obtained from Jiang et al. (2020).

### Statistical Analysis

Mass spectrometry samples were assessed at least three times independently, and all data are represented as the mean ± SD. The peak area data were preprocessed using the area normalization method, and the processed data were used for the subsequent statistical analyses. After standardization, the data fell into a specific interval [0, 1]. The heat map of gene analysis was generated using Microsoft Office Excel (Microsoft, Redmond, WA, United States).

## Results

### Metabolic Analysis of Phenolic Compounds and Expression Analysis of Related Biosynthetic Genes in Flower Buds at Different Developmental Stages

**FIGURE 2:**
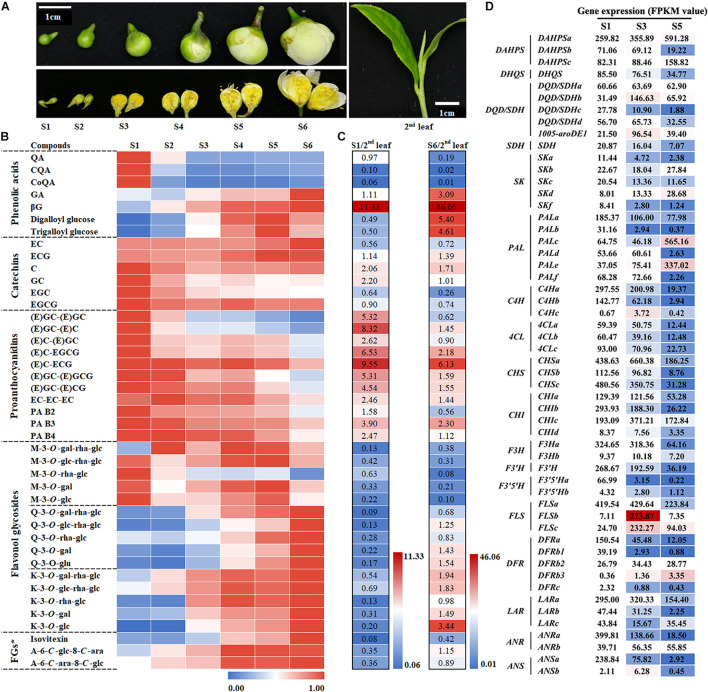
Metabolic analysis of phenolic compounds and expression analysis of related biosynthetic genes at different flower bud developmental stages. **(A)** Phenotypes of different stages of flower buds and the second leaf. S1–S6 represent developmental stages 1–6, respectively. **(B)** Characteristics of phenolic compound accumulation in different flower developmental stages. The peak area data were processed and fall into an interval of 0–1 according to the ratio. FGs*, Flavone glycosides. **(C)** Comparison of the phenolic compound accumulation between S1 or S6 and the second leaf. **(D)** Expression analysis of genes in the shikimate, phenylpropanoid, and flavonoid pathways at different flower development stages. The value represents the FPKM value. The S1 FPKM value of each gene transcript was defined as numerical value 1, and the stage 3 and S5 FPKM values were converted proportionally.

To investigate the metabolism characteristics of phenolic compounds in flower buds during the flowering process (Figure 2A), we collected flower buds at different developmental stages and used UPLC-MRM-MS system to detect differences in the types and contents of phenolic compounds among them. Given that the flower is a metamorphic branch and that the phenolic compounds in the leaves on the branch are already well understood (Jiang et al., 2013), the types and contents of phenolic compounds in the second leaf were used as references for the UPLC-MRM-MS analysis. The results revealed no significant difference in the types of phenolic compounds between tea leaves and flowers (Supplementary Table 1 and Figure 2C). Similar to leaves, flowers have the following main types of phenolic compounds: phenolic acids, catechins (flavan-3-ols), proanthocyanins (PAs), flavanols, and flavonol glycosides (FGs). Their biosynthesis involves the shikimate pathway and its subsequent phenylpropanoid and flavonoid pathways (Wang et al., 2018). Several typical derivatization reactions, such as galloylation and condensation of flavan-3-ols, glycosylation of flavone and flavonol, and hydroxylation reactions of flavonoids, enrich these compounds (Zhuang et al., 2020).

Catechins, especially galloyl type EGCG and ECG, are the main phenolic compounds in tea leaves and flowers. Because proanthocyanins are the condensation products of catechins monomers, proanthocyanin and catechin monomers share the same synthesis pathway. Heat map analysis revealed that, in addition to EC and ECG, these catechins and PAs compounds exhibit similar patterns of change in flower development—their content gradually decreases as the flower develops (Figure 2B). In addition, the proanthocyanin content of the flowers in both S1 and S6 was significantly higher than that in the second leaf, but the content of the main catechin EGCG exhibited the opposite trend. This means that the condensation reaction rate in flowers is higher than that in leaves, whereas galloylation exhibited the opposite trend.

The derivatives of gallic acid (GA) and quinic acid (QA), the main phenolic acid derivatives, are derived from different branches of the shikimic acid (SA) pathway. They exhibited completely different trends in flower development: the content of GA derivatives gradually increased with the development of the flower, whereas that of QA derivatives reached the highest level in stage 1 (Figure 2B). In the second leaves, the content of GA derivatives was greatly increased. For example, the β-glucogallin (βG) content was 46.06 times higher in the flower bud at stage 6 than in the second leaf. Given that the content of hydrolyzed tannins in flowers is high, flower buds are a suitable substitute for studying the biosynthesis of hydrolyzed tannins in tea plants.

Based on the difference in the number of hydroxyl groups in the B-ring, flavonols can be divided into monohydroxy, dihydroxy, and trihydroxy compounds. Their representative glycosides are kaempferol (K), quercetin (Q), and myricetin (M), respectively. M glucosides with trihydroxy in the B-ring display a different trend from K and Q glucosides during flower development. During flower development, the contents of K and Q glucosides display an increasing trend, especially when their content exceeds that in the second leaf in the flower bud at S6.

In summary, the increase in the content of flavonol and GA derivatives is a significant metabolic feature during flower bud development. Compared with that in the second leaf, the content of QA derivatives, M derivatives, and the main catechin EGCG in the flower bud is lower.

To further understand the differences in gene expression related to phenol metabolism during flower development, we implemented a transcriptome sequencing project by using the BGISEQ-500 platform and the flower buds at stages 1, 3, and 5 as materials and focused on the differential expression of structural genes and transcription factor genes related to the shikimate, phenylpropanoid, and flavonoid pathways. RNAseq raw data has been submitted to Sequence Read Archive (SRA,^2^), and the accession number is PRJNA760230.

The results revealed that different members of the five gene families, such as *DHD/SDH* and Phospho-2-dehydro-3-deoxyheptonate aldolase (*DAHPS*) gene families, belonging to the shikimate pathway exhibit a differential expression pattern during flower development. The functions of 39 transcripts of 13 gene families belonging to the phenylpropanoid and flavonoid pathways have been verified (Wang et al., 2018). Our results indicated that most members of the 13 gene families were highly expressed in the flower buds at stage 1 (Figure 2D). A few genes were highly expressed in flowers at stage 3 or 5, such as *PALc* and *PALe*, *C4Hc*, *FLSb*, *FLSc*, and *DFRb3*. This was also evident in the differential expression analysis of transcription factors including MYB, bHLH, and the glycosylation-related UDP-glycosyltransferase gene family (Supplementary Table 2). Notably, the gene expressions of *FLSb* and *FLSc*, which are involved in flavonol synthesis, were significantly upregulated at stage 3, which was probably related to flavonol accumulation at the different developmental stages of tea flowers. Real-time quantitative PCR was performed to verify the reliability of the transcriptometric sequencing results, and the results were consistent with the transcriptometric sequencing results (Supplementary Figure 1).

### Metabolic Analysis of Phenolic Compounds and Expression Analysis of Related Biosynthetic Genes in Various Parts of Tea Flowers

**FIGURE 3:**
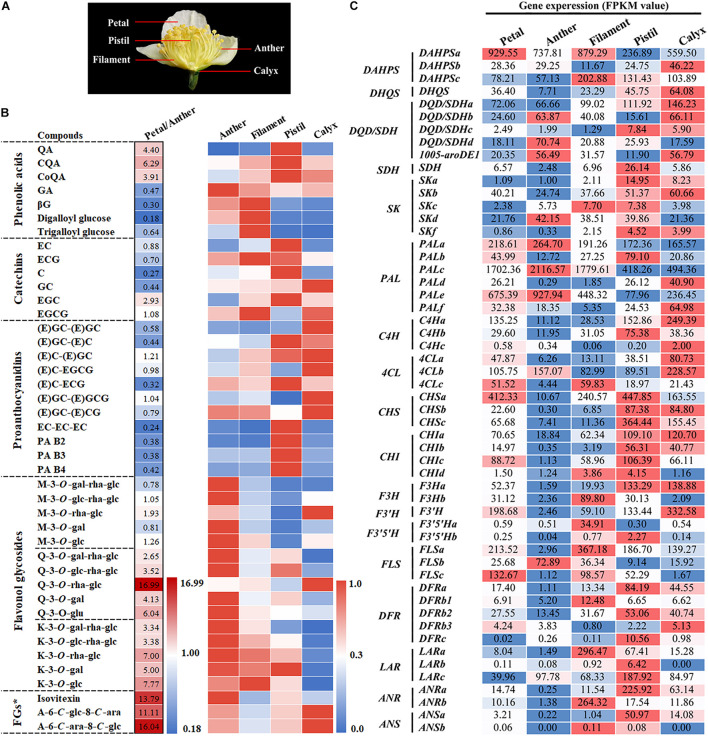
Metabolic analysis of phenolic compounds and expression analysis of related biosynthetic genes in different parts of tea flowers. **(A)** Phenotypes of different parts of the flower. **(B)** Metabolic analysis of the main phenolic compounds in different parts of tea flowers. The petal/anther data were relative peak area comparisons of the petal to the anther. The peak area data of anthers, filaments, pistils, and calyxes were processed and fell into the interval [0, 1] according to the ratio. FGs*, Flavone glycosides. **(C)** Expression analysis of genes in the shikimate, phenylpropanoid, and flavonoid pathways in different parts of the tea flower. The value represents the FPKM value.

The tea flower can be divided into petal, stamen (anther and filament), pistil (stigma and style), and calyx (Figure 3A). Among them, Petals, anther and filament are the main components of flowers, accounting for 36, 34, and 25% respectively. UPLC-MRM-MS analysis was used to detect and analyze the metabolism of polyphenols in these parts. The results indicated that the polyphenol content of various parts of the flower bud varied greatly (Figure 3B). The contents of flavonol and flavone glycosides in petals and anthers were significantly higher than that in other parts of the flower bud, and their contents were the highest in petals. Thus, petals and anthers were determined to be the main sources of flavonols in tea flowers at stage 6. Moreover, filaments were the main source of GA derivatives in tea flowers at stage 6. Because the content of GA derivatives and gallic acylated catechins, including EGCG and ECG, was higher in filaments than in other flower bud parts, we speculate that the galloylation catalyzed by the SCPL enzyme in filaments was higher than that in other parts.

The content of B-ring dihydroxycatechins and their polymer was higher in the pistil than in other flower bud parts. However, the contents of monomers and polymers of B-ring trihydroxy compounds such as EGC, GC, and EGCG and M glycoside compounds were lower in pistils than in the other parts. This means that the predominant F3′5′H-catalyzed B-ring trihydroxylation reaction in tea leaves was weaker in pistils. Catechin accumulation characteristics in the calyx were opposite to in the pistil. Similar to leaves, the calyx was observed to accumulate more B-ring trihydroxy catechin monomers and their polymers.

In conclusion, as a specialized or metamorphic branch, the accumulation of phenolic compounds in various parts of the tea flower exhibits differing characteristics, which also means that the flower is a good material for studying the different branches of biosynthesis of phenolic compounds of tea plants.

To further understand the differences in gene expression related to phenol metabolism in different parts of the flower bud, we analyzed the transcriptome information of various parts of flower buds. According to the transcriptomic analysis of different developmental stages of tea flowers, we analyzed the genes related to the shikimate, phenylpropanoid, and flavonoid pathways. The results are illustrated in Figure 3C. *DFR*s, *LAR*s, *ANR*s, and other genes were highly expressed in the pistil and calyx; such expression was related to the accumulation of catechins, proanthocyanins, and other substances in the pistil and calyx. *F3H*, *FLS*s, and other genes related to the synthesis of flavonol were highly expressed in anthers and filaments; this was related to the accumulation of flavonol in flower petals, anthers, and filaments. Notably, *FLSb* exhibited a particularly high expression pattern in anthers, suggesting that *FLSb* may be closely related to anther development.

### Correlation Between Flavonols and Tea Flower Fertility

**FIGURE 4:**
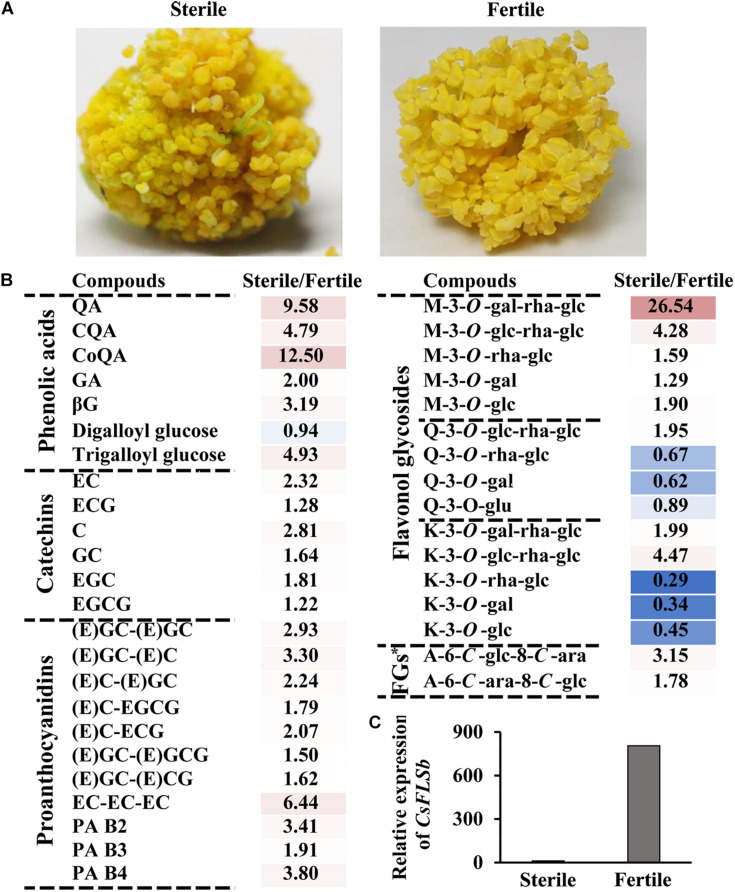
Characteristics of phenolic compound accumulation in the anthers of hybrid sterile and fertile tea flowers and *CsFLSb* gene expression in hybrid sterile and fertile flowers. **(A)** Anther phenotypes of the hybrid sterile flower with no pollen and fertile flowers. **(B)** Metabolic analysis of phenolic compounds between hybrid sterile anther and fertile anther. FGs*, Flavone glycosides. **(C)** *CsFLSb* expression in hybrid sterile and fertile flowers; the histogram shows the FPKM values.

The results described in the previous section suggest that the anther has a high flavonol content. This raises the question of whether flavonols are closely related to the development of anthers. To answer this question, we compared phenolic content between fertile flowers and hybrid sterile flowers without pollen (Figures 4A,B). In addition to some K and Q glycosides, UPLC-MRM-MS analysis revealed that the contents of most phenolic compounds, including catechins, proanthocyanins, phenolic acids, flavone glycosides, and M derivatives in sterile flowers, were higher than those in fertile flowers. For example, the contents of Kaempferol-3-*O*-glucoside (K-3-*O*-glc) and Kaempferol-3-*O*-rutinoside (K-3-*O*-rha-glc) in sterile flowers are only 45.2 and 29.0% of the contents in fertile flowers, respectively. This result suggests that K and Q glycosides are probably related to the development of the anther. Moreover, compared with fertile flowers, *CsFLSb* expression in sterile flowers was extremely low.

### Functional Differences Between *CsFLSa*, *CsFLSb*, and *CsFLSc*

**FIGURE 5:**
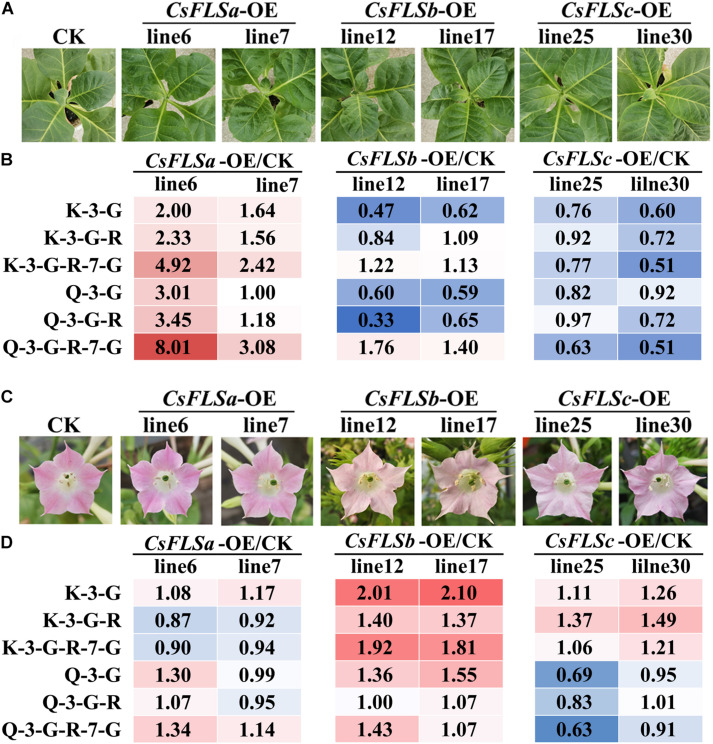
Flavonol accumulation characteristics in *CsFLS*s transgenic tobacco leaf and flower. **(A)** Phenotypes of CK and *CsFLSa*, *CsFLSb*, and *CsFLSc* transgenic tobacco leaves. **(B)** Relative accumulation of kaempferol and quercetin glycoside extracts from CK and *CsFLS*s tobacco leaves. **(C)** Phenotypes of CK and *CsFLSa*, *CsFLSb*, and *CsFLSc* tobacco flowers. **(D)** Relative accumulation of kaempferol and quercetin glycosides of CK and *CsFLS*s tobacco flowers. CK, empty vector control.

Three flavonol synthase genes—*CsFLSa*, *CsFLSb*, and *CsFLSc*—were analyzed to determine their function in flavonol synthesis. The enzymatic properties of the recombinant protein of these three genes have been investigated (Jiang et al., 2020). However, the differences in their function in plants have not been thoroughly studied. By using the pCB2004-*Agrobacterium tumefaciens* expression system, we expressed *CsFLSa*, *CsFLSb*, and *CsFLSc* in tobacco and obtained stable transgenic tobacco plants. The growth status of these transgenic plants was consistent with that of the control transgenic plants (CK; Figure 5A). Compared with CK tobacco flowers, the flowers of the three transgenic tobacco plants heterologously expressing *CsFLSb* and *CsFLSc* exhibited changes in their characteristics (Figure 5C). Compared with the pink CK tobacco flower, the color of *CsFLSb*-OE and *CsFLSc*-OE tobacco flowers was lighter—almost white, whereas that of *CsFLSa* transgenic tobacco flower remained seemingly unchanged (Figure 5C).

The accumulation of flavonols in CK and three transgenic tobacco flowers and the first and second leaves was detected with UPLC-MRM-MS. The characteristics of flavonol accumulation in transgenic tobacco leaves and flowers differed (Figure 5B). Compared with CK, the flavonol content increased in the leaves of *CsFLSa*-OE plants and decreased in those of *CsFLSb*-OE and *CsFLSc*-OE plants. However, the flavonol content of the *CsFLSa* tobacco flower exhibited no distinct change, whereas that of *CsFLSb*-OE and *CsFLSc*-OE tobacco flower flavonol content, especially K flavonol glycosides, increased significantly; and Q flavonol glycoside content decreased in *CsFLSc*-OE tobacco flowers (Figure 5D).

In summary, *CsFLSa*, *CsFLSb*, and *CsFLSc* regulate flavonol accumulation in transgenic tobacco in different parts. Accordingly, we speculate that these genes have different functions in tea plants.

## Discussion

Flavan-3-ols with a C6-C3-C6 structure are the main compounds accumulated in leaves of tea plants, accounting for 70% of the total polyphenols (4). Second only to that of flavan-3-ols, flavonol glycoside content in tea leaves can reach >2% (Wu et al., 2016). Both flavan-3-ols and flavonol glycosides are directly related to the bitterness and astringency of tea beverages (Zhuang et al., 2020). Therefore, their biosynthesis has attracted much attention from scientists. The main pathways of favan-3-ols and flavonol glycosides synthesis are the same as those in Arabidopsis and other model plants. They all come from the shikimate pathway and its downstream phenylpropanoid and flavonoid synthesis pathways (Jiang et al., 2013; Figure 1).

Each step in the shikimate, phenylpropanoid, and flavonoid synthesis pathways is controlled by multiple gene families (Wang et al., 2018). This means that these pathways have network regulation characteristics. Phenolic acids, including SA and GA derivatives, mainly come from the SA pathway located upstream of the phenylpropanoid pathway (Figure 1). Huang et al. (2019) reported that four 3-dehydroquinate dehydratase/shikimate dehydrogenase (*DQD/SDH*) genes in tea plants are responsible for the synthesis of SA and GA, respectively. In particular, the SDH enzyme, a bifunctional enzyme, controls the synthetic metabolic flow of GA, QA, and SA in plants. The results presented in Figure 2 show that the accumulations of QA and GA derivatives exhibit very different trends, a finding which may be attributable to the differential expression of these gene family members.

The biosynthesis of flavan-3-ols or flavonol glycosides in various organs of tea plants can be controlled by different homologous genes of the same gene family. Because the types of phenolic compounds accumulated in different parts of the tea flower are highly different (Figure 2), floral organs may be good materials for studying the network regulation of phenolic compound biosynthesis.

Many studies have attempted to identify flavonols and their biosynthesis regulation in tea plants (Wu et al., 2016; Jiang et al., 2020). However, their physiological significance in tea plants remains unclear. Given the antioxidant function of phenolic compounds *in vitro* (Jiménez-Zamora et al., 2016; Zeng et al., 2017), it is speculated that phenolic compounds in plants can also participate in the plant’s antiepidemic response through their antioxidant capacity. In many model plants, phenolic compounds, including anthocyanins, phenolic acids, lignin, flavonols, and proanthocyanins, participate in the physiological processes of plant growth, flowering and fruiting, and resistance to biotic and abiotic stresses (Muro-Villanueva et al., 2019; Shi et al., 2019; Su et al., 2020). This article found that not only the leaves, but also the flowers of tea plants accumulate high levels of phenolic compounds (Figure 2). So, is the so highly accumulated phenolic compounds in tea flowers related to its development or resistance?

Flavonols are involved in the development of floral organs and flower fertility (Mo et al., 1992; Napoli et al., 1999). *CHS* gene knockout in petunias and *FLS* gene silencing in tobacco can suppress the whitening of anthers, induce male sterility, and influence other traits (Napoli et al., 1999; Mahajan et al., 2011). Compared with wild-type *A. thaliana*, that with low flavonol accumulation significantly inhibits pollen tube growth (Schiefelbein et al., 1993). The pollen germination and pollen tube elongation can be restored by adding low-concentration flavonol to the germination medium (Ylstra et al., 1992; Pollak et al., 1995). In this study, we also found a correlation between flavonols and anther growth (Figure 4). During flower development, the content of flavonol K and Q glycosides gradually increased (Figure 2). Mass spectrometry detection of materials in different parts of the flower revealed that most flavonol glycosides mainly accumulated in petals and anthers (Figure 3). The content of K and Q glycosides in sterile flower anthers was lower than that in fertile flower anthers (Figure 4). Our transcriptomic analysis (Figures 2–4) and the previous studies (Chen et al., 2019) screened out flavonol pathway genes related to flower development and fertility. *CsFLSb* changed significantly during flowering, and its expression was high in anthers in fertile flowers and extremely low in sterile flowers. These results indicate that *CsFLSb* played an essential role in the regulation of anther development by affecting flavonol synthesis during flower development. Our study findings on phenol accumulation, together with transcriptomic analysis, help to reveal the mechanism of sterility in tea plants. However, molecular data are limited for tea plants. Future studies should attempt to elucidate the other aspects mechanisms. Our metabolome and transcriptome analysis results help to reveal the mechanism of tea flower sterility. However, these landscape data alone are not enough, and further mechanism studies are needed in the future.

Flavonol synthases are key genes in flavonol synthesis. Notably, in the leaves of tea plants, the expression of *FLSa* is considerably high and that of *FLSb* and *FLSc* is relatively low (Wang et al., 2018). However, *FLSb* and *FLSc* expression in the flowers in stage 3 was 38.5 times and 9.4 times higher, respectively, than that in S1 (Figure 2). The function of *CsFLS* genes has been studied using enzymology and transgenic tobacco methods. An enzymological analysis revealed three recombinant *CsFLS* proteins that can catalyze dihydroflavonols to form flavonols (Jiang et al., 2020). In this study, we observed that tobacco overexpressed with *CsFLSa*, *CsFLSb*, and *CsFLSc* showed the opposite trend of accumulating flavonol (Figure 5). This result further proved that *CsFLSb* and *CsFLSc* rather than *CsFLSa* are responsible for the synthesis of flavonol glycosides in the flowers of tea plants.

## Data Availability Statement

The data presented in the study are deposited in the Sequence Read Archive repository, accession number PRJNA760230.

## Author Contributions

LG and TX conceived and designed the study. YS, XJ, LG, and TX drafted the manuscript. YS performed the experiments. YS and XJ analyzed the data. LC provided some plant materials and transcriptomic data. ZF, W-WL, SL, and YQ assisted with experiments, materials, and analytical tools. YL reviewed and edited the manuscript. All authors read and approved the final version of the manuscript.

## Conflict of Interest

The authors declare that the research was conducted in the absence of any commercial or financial relationships that could be construed as a potential conflict of interest.

## Publisher’s Note

All claims expressed in this article are solely those of the authors and do not necessarily represent those of their affiliated organizations, or those of the publisher, the editors and the reviewers. Any product that may be evaluated in this article, or claim that may be made by its manufacturer, is not guaranteed or endorsed by the publisher.

## Footnotes

1
http://tpia.teaplant.org

2
https://www.ncbi.nlm.nih.gov/sra/?term=PRJNA760230

## Supplementary Material

Supplementary Figure 1 Verification of the relative expression levels of genes by qRT-PCR. Expression patterns of parts of structural genes in S1, S3, S5 flowers involved in phenylpropanoid and flavonoid pathways by qRT-PCR, including **(A)** C4Ha, **(B)** 4CLb, **(C)** CHIc, **(D)** F3Ha, **(E)** F3′H, **(F)** FLSb, **(G)** LARc, **(H)** ANRa, and **(I)** ANRb.
