# Supplementary figures and images for: Functional Analyses of Flavonol Synthase Genes From Camellia sinensis Reveal Their Roles in Anther Development
Source: Front Plant Sci. 2021 Oct 1;12:753131. doi: 10.3389/fpls.2021.753131 (PMC8517536; doi:10.3389/fpls.2021.753131)

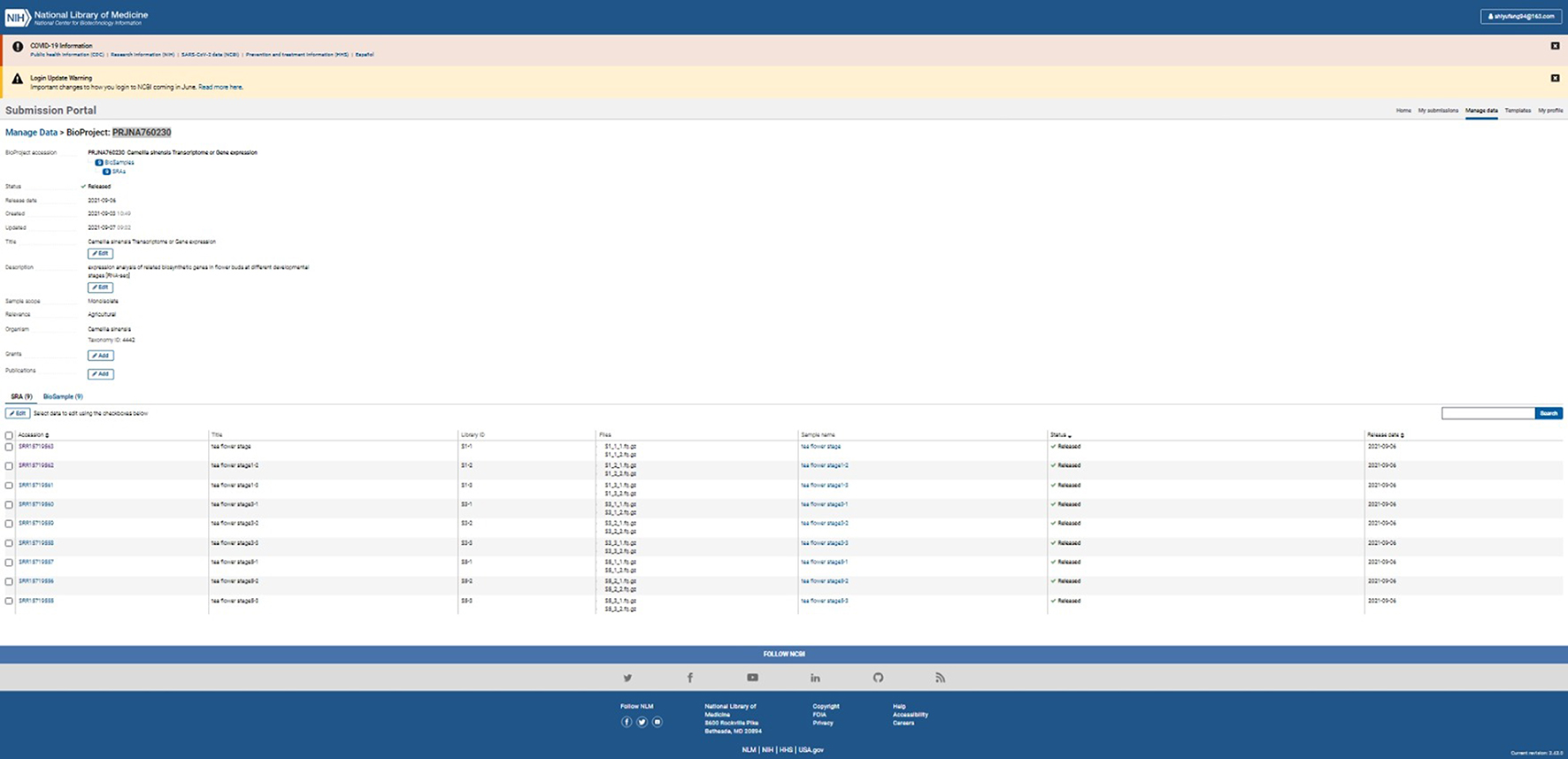

Supplement: Supplementary Figure 1 — Verification of the relative expression levels of genes by qRT-PCR. Expression patterns of parts of structural genes in S1, S3, S5 flowers involved in phenylpropanoid and flavonoid pathways by qRT-PCR, including (A) C4Ha, (B) 4CLb, (C) CHIc, (D) F3Ha, (E) F3′H, (F) FLSb, (G) LARc, (H) ANRa, and (I) ANRb. [file Image_1.jpeg]
